# Budget impact analysis of the use of extended half-life recombinant factor VIII (efmoroctocog alfa) for the treatment of congenital haemophilia a: the Italian National Health System perspective

**DOI:** 10.1186/s12913-018-3398-x

**Published:** 2018-08-02

**Authors:** Valentina Lorenzoni, Isotta Triulzi, Giuseppe Turchetti

**Affiliations:** Institute of Management, Scuola Superiore Sant’Anna, Piazza Martiri della Libertà n. 33, Pisa, Italy

**Keywords:** Budget impact, Haemophilia, Recombinant products, rFVIIIFc

## Abstract

**Background:**

Congenital haemophilia A (HA) is a rare, inherited, life-long bleeding disorder characterised by prolonged or spontaneous bleeding due to the lack of clotting factor VIII (FVIII) in the body. Treatment for HA involves FVIII replacement therapy and poses great economic burden to National Health Systems and to society. Availability of novel products as extended half-life clotting factor products might change treatment approches and their economic evaluation is essential for an informed treatment choice. Accordingly the objective of the present work is to analyse the economic impact of using efmoroctocog alfa (recombinant factor VIII-Fc fusion protein, rFVIIIFc) for the treatment of children and adults with severe congenital haemophilia A (HA).

**Methods:**

A budget impact analysis was performed to estimate the economic impact of the introduction of rFVIIIFc in the market-mix of products for the treatment of HA. The analysis condidered a 3-year time horizon and the Italian National Health System (INHS) perspective. The model estimated drug costs associated with the treatment of HA in the current scenario - representing the marketplace forecast for the time period of interest assuming that rFVIIFc is not introduced - and a new scenario, assuming that rFVIIIFc is available in the market. The size of the target population was calculated using epidemiological national data. Univariate one-way sensitivity analyses and scenario analyses were performed.

**Results:**

Overall 3-year costs of treating the HA population in the current scenario were 555,277,691 Euro for the INHS. With the introduction of rFVIIIFc, the costs were reduced to 541,897,466 Euro suggesting potential savings to the INHS of 13,380,255 Euro. Results were consistent at variation of most of the model’s parameters; only in case of lower dosage of conventional products and higher dosage of rFVIIIFc, costs for the INHS increased, in both cases, of about 20 million Euro.

**Conclusions:**

The use of rFVIIIFc for the treatment of HA has been recently approved by the Italian Medicines Agency (AIFA) and this is the first study estimating the financial impact of this new therapeutic alternative in the Italian context. The analysis suggests that rFVIIIFc use does not result in higher expenditure for the INHS.

## Background

Congenital haemophilia A (HA) is an inherited bleeding disorder caused by deficiency of factor VIII (FVIII) occurring at a rate of approximately 10–20 in every 100,000 live births [1–3].

HA patients suffer from a high risk of spontaneous and traumatic bleeding into joints, muscles and soft tissues requiring the infusion of the deficient coagulating factor.

Treatment for HA involves deficient clotting factor (FVIII) replacement therapy administered either prophylactically, to prevent bleeding events, or on-demand, that is on an “as needed basis” to treat active bleeding. In Italy, on-demand treatment is mainly reserved for patient with mild HA [4].

Current conventional prophylactic regimens require treatment three times weekly or every other day for the treatment of severe haemophilia A to prevent patients’ FVIII plasma activity levels from falling below critical levels (usually 1%). The short half-life of FVIII products can be a barrier for prophylaxis, in particular for children necessitating more frequent infusions because of the higher clearance and the shorter half-life of drugs in these patients compared to adults [5, 6].

In the last few years, the development of a new generation of FVIII products with improved pharmacokinetic profile and less immunogenic characteristics, led to outstanding results. In fact, these medicines may improve the management of the patients and their quality of life by reducing the burden of frequent intravenous injections, the need for central venous line in children, and the loss of adherence typical in adolescents. Theremore, the new extended half-life FVIII concentrates allow to do physical activity and to manage surgical procedures with few injections and low factor consumption [7, 8].

Among the new generation of FVIII, a protein composed of a single molecule of recombinant FVIII (rFVIII) fused to the Fc domain of IgG1 (rFVIIIFc), has recently been developed. Because of its molecular structure, rFVIIIFc shows a longer life when compared to other conventional factor VIII. Once infused, the Fc domain of these fusion proteins binds to the neonatal receptor (FcRn) in the endosomes, expressed in many cells types, including endothelial cells. Given that the receptor FcRn protects IgG from degradation, Fc-fusion protein prolongs the half-life of the drug utilizing the IgG recycling pathway, delaying the lysosomal degradation and cycling them back into the blood circulation [9].

Evidence showed that rFVIIIFc has approximately 1.5-fold longer half-life and a 33% lower clearance [5, 7] than the conventional rFVIII, thus allowing for fewer infusions as well as greater protection and individualisation of therapy than is currently possible with conventional products [7, 10–12]. Although more studies evaluating the immunological profile are needed, currently available evidence suggests that rFVIIIFc does not increase the immunogenicity [5, 7, 10]; data about tolerance and safety are also encouraging [8, 13].

The introduction of recombinant factor VIIIs and the improvements in clinical practice in the ‘70s ameliorated the management of HA patients, allowing prevention of bleeding, improved long-term outcomes and increased life expectancy from 40 years to 60–70 years old today [4], at the price of increased disease associated costs [14].

Available studies have demonstrated that factor replacement therapy accounts for 50–90% of the total direct healthcare costs in patients with haemophilia, depending on the severity of the disease [4, 15].

Since concerns about the cost of orphan medicines exist amongst health policy makers at the European, country and local level, and also there is little published evidence about current or future budget impact of orphan medicines in Europe, the aim of this study is to contribute to evidence generation in the field of the economic impact associated with orphan drugs by describing and reporting results of a budget impact analysis (BIA) to estimate the potential financial impact following the introduction of Elocta® (rFVIIIFc) in the market-mix of products currently available for prophylaxis treatment of paediatric and adult HA patients from the Italian National Healthcare System (INHS) perspective.

## Methods

### Analytical framework

A budget impact analysis was performed to evaluate the potential financial impact deriving from the introduction of rFVIIIFc in the market-mix of available treatments for congenital haemophilia A. The analysis considered the perspective of the Italian National Health System and was conducted over a 3-years time horizon.

**Fig. 1:**
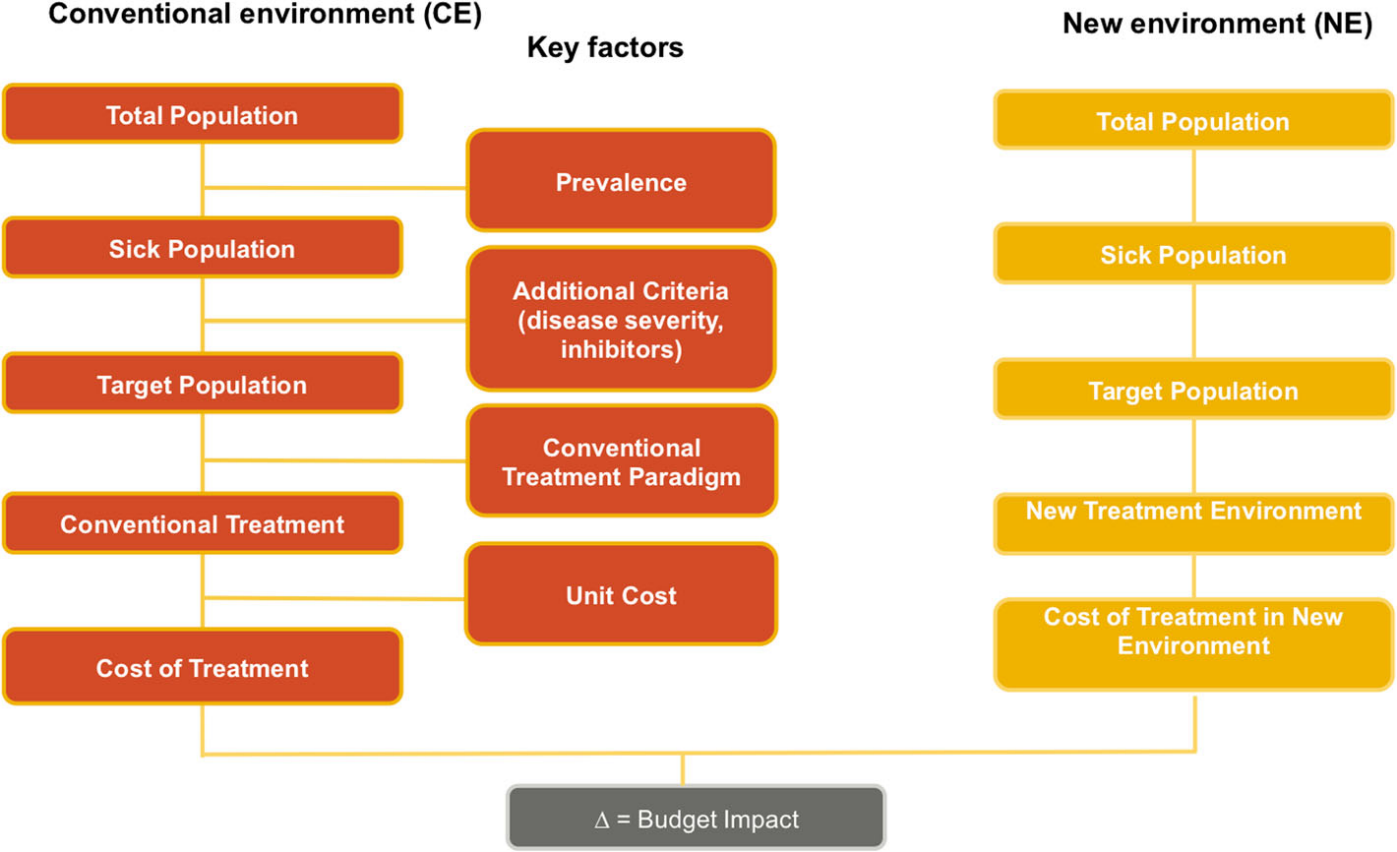
Schematic representation of the Budget Impact Model

A budget impact model (BIM) was developed as a Microsoft Excel® macro-enabled workbook to evaluate the incremental budget impact of introducing rFVIIIFc for prophylactic treatment of severe haemophilia A in paediatric and adult patients (Fig. 1). The incremental budget impact was calculated by subtracting the cost of the new treatment mix, in which rFVIIIFc is reimbursed, from the cost of the conventional treatment mix without rFVIIIFc. Full details of all assumptions used to develop the base case analysis are provided in Table 1.

**Table 1:** Assumption used to develop the base case analysis

Assumption	References
The target population is represented by patients with severe haemophilia A	Product indication
The use of rFVIIIFc is considered only for prophylaxis treatment	Product indication
Product consumption related to the use of drugs for surgery is not included in the analysis because no difference between the two scenario are hypothesized	Assumption based on the opinion of clinical experts
Patients with inhibitors are excluded from the analysis because deserving particular treatment different from those analysed in the present analysis	Product indication
Paediatric patients are exposed only to prophylaxis regimen, no on-demand treatment is considered for these patient according to current clinical practice in Italy	Assumption based on the opinion of clinical experts
According to current clinical practice in Italy, paediatric patients are not exposed to treatment with plasma derived products	Assumption based on the opinion of clinical experts
Age cut-off to differentiate treatment for paediatric and adults patients is considered equal to 12 years old	Assumption based on the opinion of clinical experts, [7]
An equal treatment schedule is considered for both plasma derived and conventional recombinant products	Assumption based on the opinion of clinical experts
The incidence of spontaneous bleeding is equal between patients treated with plasma derived and conventional recombinant products	Assumption based on the opinion of clinical experts
The consumption of products for the management of spontaneous bleeding is equal between patients treated with plasma derived and conventional recombinant products	Assumption based on the opinion of clinical experts
For all products compliance with prophylaxis treatment is considered to be 100%	Assumption based on the opinion of clinical experts
Percentage of patients switching to rFVIIIFc is considered to do not differ among the diverse products	Assumption based on the opinion of clinical experts

### Target population

The target population is represented by adult and paediatric male patients with severe congenital haemophilia A receiving prophylactic therapy. Patients with inhibitors were not included in the analysis.

The size of the population for the first year was calculated applying data about prevalence of disease in males, severity distribution of the disease, treatment regimen and prevalence of inhibitors according to severity of disease - obtained from data published in the National Registry of Blood Disorders [16] - to the resident Italian population, distributed by age and gender [17] (Table 2).

**Table 2:** Parameter used in the base-case

Type of parameter	Base Case value	References
*Demographics*		
Population	60,795,612	[18]
Male in the health plan (% of total)	48.5%	[17]
Paediatric in the health plan (% of total)	11.9%	[17]
Annual population growth	0.5%	[18]
Paediatric weight (Kg)	18.5	[21]
Adult weight (Kg)	70.6	[22]
*Epidemiology*		
HA prevalence in male	12.7 per 100,000	[16]
Severity distribution of the disease		
Mild	39.7%	[16]
Moderate	14.1%	
Severe	46.2%	
Patients without inhibitors		
Mild	99.1%	[16]
Moderate	96.3%	
Severe	81.6%	
Treatment regimen among adults		
On demand	42.8%	[16]
Prophylaxis	55.2%	
Treatment regimen among paediatrics		
On demand	0%	Assumption based on the opinion of clinical experts
Prophylaxis	100%	
*Costs*		
rFVIIIFc unit cost (€/IU)	0.72	[25]
Advate® unit cost (€/IU)	0.65	[25]
Kogenate® unit cost (€/IU)	0.69	[25]
Refacto® unit cost (€/IU)	0.69	[25]
Helixate® unit cost (€/IU)	0.69	[25]
Recombinate® unit cost (€/IU)	0.60	[25]
Plasma-derived unit cost (€/IU)	0.60	[25]

An annual population growth equal to 0.50% was assumed considering the average national growth rate from the period 2009 to 2015 [18].

### Conventional and new treatment mix

The conventional treatment mix was modelled considering commonly used recombinant or plasma-derived FVIII products in Italy and their market shares at the time of the analysis.

Assuming the annual consumption of plasma-derived and recombinant products (equal to 21 and 79%, respectively [13]) as proxy of the market share of the two classes of products, then the percentage of total market sales for the different products was applied within each class.

According to the Italian market at the time of the analysis, Advate®, Kogenate®, Helixate NexGen®, ReFacto AF® and Recombinate®, were considered among recombinant FVIII products, and their market shares among the class of recombinant products are reported in Table 3.

**Table 3:** Market shares of conventional recombinant products for paediatric and adult patients

	Paediatrics	Adults
Advate®	30%	24%
Kogenate®	29%	23%
Refacto®	23%	18%
Helixate®	17%	13%
Recombinate®	1%	1%
Plasma-derived products	–	21%

The treatment mix in the new environment (NE) following the introduction of rFVIIIFc was modelled considering the addition of this product to already available recombinant factors. rFVIIIFc was assumed to progressively gain market sales from conventional factor VIIIs proportionally to their current market shares.

In the base-case scenario the uptake rate for rFVIIIFc was assumed to be 10% in the first year, increasing to 15 and 20% in the second and third year respectively, according to the estimates of the marketing authorisation holder (Table 4).

**Table 4:** Uptake-rate of the different products according to population and year of the analysis

	Year 1	Year 2	Year 3
Paediatrics			
rFVIIIFc	10%	15%	20%
Advate®	27%	25.5%	24%
Kogenate®	26.1%	24.7%	23.2%
Refacto®	20.7%	19.6%	18.4%
Helixate®	15.3%	14.5%	13.6%
Recombinate®	0.9%	0.9%	0.8%
Plasma-derived products	–	–	–
Adults			
rFVIIIFc	10%	15%	20%
Advate®	21.6%	20.4%	19.2%
Kogenate®	20.7%	19.6%	18.4%
Refacto®	16.2%	15.3%	14.4%
Helixate®	11.7%	11.1%	10.4%
Recombinate®	0.9%	0.9%	0.8%
Plasma-derived products	18.9%	17.9%	16.8%

### Resource use and costs

Only direct costs of medication were considered in the analysis. The model calculated the overall direct treatment costs of the two environments (new environment, NE, and conventional environment, CE) by multiplying the volume of the different FVIII agents consumed in each environment times the unit costs of each FVIII product. Product consumption for the different alternatives was estimated on the basis of available evidence and considering the use of products associated both to the prophylaxis treatment and to the management of bleeding episodes. All the inputs used in the model and the assumptions used were discussed and validated with a panel of seven clinicians selected among Italian specialists with extensive experience in the treatment of HA patients, health economists and statisticians. Clinicians were asked to revise data used in the model and to solve eventual uncertainty about the source of data to be used and were also asked to discuss the assumptions to be used for parameters for which no or insufficient evidence was available at the time of the analysis; data were collected as a part of an advisory board discussion.

In particular, given a lack of data in the scientific literature, for plasma-derived and conventional recombinant therapy available in the market, the experts suggested to assume an equal treatment schedule. A median dosage of 43 UI/kg every 72 h in adults and 33.8 UI/kg every 3.07 days in paediatrics, were therefore assumed based on data reported for the prophylaxis arm by Valentino et al. [19] and Blanchette et al. [20], respectively.

Median dosage and the frequency of infusion indicated in the A-LONG [10] and the Kids A-LONG [7] studies were used for rFVIIIFc (Table 5).

**Table 5:** Parameters used for quantifying product consumption associated to prophylaxis treatment and to the resolution of bleeding events in pediatric and adult patients per week

	Prophylaxis treatment			Treatment for the resolution of bleeding		
	Median dosage (IU/Kg)	Num. of administration per week	References	Annual bleeding events	Median dosage (IU/kg)	References
Paediatric patients						
rFVIIIFc	44.05	2	[7]^a^	1.96	54.9	[7]
Conventional products	33.8	3.07	[20]^b^	4	46.6	[20]
Adult patients						
rFVIIIFc	38.95	2	[10]^c^	1.66	31.32	[23]^e^
Conventional products	43.0	2.3	[19]^d^	3.84	34.5	[20, 24]^f^

^a^The study reported a median weekly dose of 88.11 IU/Kg and a median dose per infusion equal to 44.05 IU/Kg, the median number of administration per week was obtained dividing the median weekly dose by the median dose per infusion

^b^The study reported a median weekly dose of 103.8 IU/Kg and a median dose per infusion equal to 33.8 IU/Kg, the median number of administration per week was obtained dividing the median weekly dose by the median dose per infusion

^c^The study reported a median weekly dose of 77.9 IU/Kg and a median dose per infusion equal to 47.2 IU/Kg, the median number of administration per week was obtained dividing the median weekly dose by the median dose per infusion

^d^Among 141 subjects treated with individualized and standard prophylaxis and enrolled in the A-LONG study a total of 301 bleeding events were recorded over 67 weeks, annual number of bleeding for a single subjects was thus obtained as the ratio of number of bleeding events and person-days (301/(67*7*141))*365.25

^e^The study reported a median dose per infusion equal to 43.0 UI/Kg during the individualized prophylaxis regimen. In the model the median dose and average dose are equivalent

^f^Valentino et al.[19] reported a total of 245 bleeding events over 23,282 person-days, data were adapted to 1 year multiplying the bleeding rate observed in the study by 365.25 [(245/23,282)*365.25]

Products consumption for prophylaxis treatment over one year was then obtained multiplying median dosage, frequency of administration and the mean weight of paediatric and adult patients in Italy [21, 22].

The product consumption associated with the treatment of a bleeding event was estimated considering the median number of bleeding events over 1 year (expressed as Annualized Bleeding Rate, ABR) and the median dose needed to treat the bleeding. Data reported in the rFVIIIFc Summary of Product Characteristics (SmPC) [23] and in the Kids A-LONG study were used for rFVIIIFc. For all the other products, data on bleedings were obtained from Blanchette et al. [20] for paediatric patients and from Valentino et al. and Tarantino et al. [19, 24] for adult patients (Table 5). ABR over 1-year and the median dose needed to treat the bleeding were assumed not differ among the different medicines.

The model was developed considering the prices listed in the Official Gazette [25] for each conventional factor VIII, Table 2. The ex-factory prices considered in the base-case analysis were: 0.65 Euro/IU for Advate®, 0.69 Euro/IU for Kogenate®, 0.69 Euro/IU Helixate NexGen®, 0.69 Euro/IU ReFacto AF® and 0.60 Euro/IU both for Recombinate® and plasma-derived products.

For rFVIIIFc a price of 0.72 Euro/IU was considered in the base-case analysis.

No discount rate was applied according to the recommended principle and good practice for performing a BIA [26].

### Sensitivity analysis

Univariate sensitivity one-way analysis was performed to assess the robustness of the results from the base-case. The major parameters used in the analysis were varied once as detailed in Table 6.

**Table 6:** Base-case parameters and values used in sensitivity analysis

	Base-case	Sensitivity analysis	
Prevalence: ±30%			
	*12.7 per 100,000*	6.4 per 100,000	19.1 per 100,000
Patients treated with prophylaxis: ±25%			
	*55%*	41%	69%
rFVIIIFc uptake rate: −50%; + 25%			
Year 1	*10%*	5%	12.5%
Year 2	*15%*	7.5%	18.8%
Year 3	*20%*	10%	25%
rFVIIIFc dosage (IU/Kg): ±30%			
Paediatric patients	*44.1*	30.8	57.3
Adult patients	*38.95*	27.3	50.6
Dosage of conventional products (IU/Kg): ±30%			
Paediatric patients	*33.8*	23.7	43.9
Adult patients	*43*	30.1	55.9
Products cost (Euro/IU): −15% rFVIIIFc;– 15% conventional products			
rFVIIIFc	*0.72*	0.61	0.72
Advate®	*0.65*	0.65	0.55
Kogenate®	*0.69*	0.69	0.59
Refacto®	*0.69*	0.69	0.59
Helixate®	*0.69*	0.69	0.59
Recombinate®	*0.60*	0.60	0.51
Plasma-derived product	*0.60*	0.60	0.51

According to the budget impact analysis-principles of good practice and recent suggestion for the conducting of economic evaluation in the field of haemophilia [26, 27], rather than selecting arbitrarily values for parameters, expert opinion and literature data were used to determine ranges of parameters to be tested in the sensitivity analysis.

In particular, the range of values used for the prevalence of the disease and the percentage of subjects treated on-demand versus prophylaxis were based on the opinion of the clinical experts involved in the study; similarly, the range of values for FVIIIs’ costs and the uptake-rate for rFVIIIFc were determined based on the opinion of market specialists. Variations of drug dosage were assumed based on evidence from the ASPIRE study [28] indicating a median dosage reduction for rFVIIIFc equal to 37%, and using a conservative approach.

Results of the sensitivity analysis are presented in a Tornado diagram showing the potential impact on the base-case results of uncertainty about the main parameters used in the model.

### Scenario analysis

Different scenario analyses were performed varying some of the assumptions used in the base-case analysis.

Particularly:

- Scenario Analysis 1 was conducted considering the possibility to expand the treated population by including in the target population subjects with moderate disease;
- Scenario Analysis 2 considered the possibility to switch from on-demand to prophylaxis treatment for both plasma-derived products and recombinant factors VIII (for simplicity a 5% switch rate was assumed).

## Results

In the base-case analysis the 3-year costs associated to the CE and NE were estimated to be 555,277,691 Euro and 541,897,466 Euro respectively, indicating savings for the INHS equal to 13,380,225 Euro when rFVIIIFc is available in the Italian market.

As detailed in Table 7, savings induced by the introduction of rFVIIIFc among the products for treating haemophilia A steadily increased over the years due to the greater number of haemophilic patients progressively receiving rFVIIIFc as a therapy. At the end of the three years of analysis the drug expenditure was reduced by 3.2%.

**Table 7:** Budget impact

	Overall num. Patients	Num. patients treated with efmoroctocog alfa	Cost CE (Euro)	Cost NE (Euro)	Savings (Euro)	% Savings
Year 1						
Pediatrics	169	17	11,812,558	11,686,364	126,194	1.1%
Adults	691	69	172,352,752	169,523,751	2,829,001	1.6%
Total	860	86	184,165,311	181,210,115	2,955,196	1.6%
Year 2						
Pediatrics	170	26	11,871,934	11,681,691	190,243	1.6%
Adults	694	104	173,219,079	168,954,248	4,264,831	2.5%
Total	864	130	185,091,013	180,635,939	4,455,074	2.4%
Year 3						
Pediatrics	171	34	11,931,608	11,676,676	254,932	2.1%
Adults	698	140	174,089,760	168,374,735	5,715,025	3.3%
Total	869	174	186,021,368	180,051,411	5,969,957	3.2%
Total						
Pediatrics	510	77	35,616,100	35,044,731	571,369	1.6%
Adults	2083	313	519,661,591	506,852,735	12,808,856	2.5%
Total	2593	390	555,277,691	541,897,466	13,380,225	2.4%

**Fig. 2:**
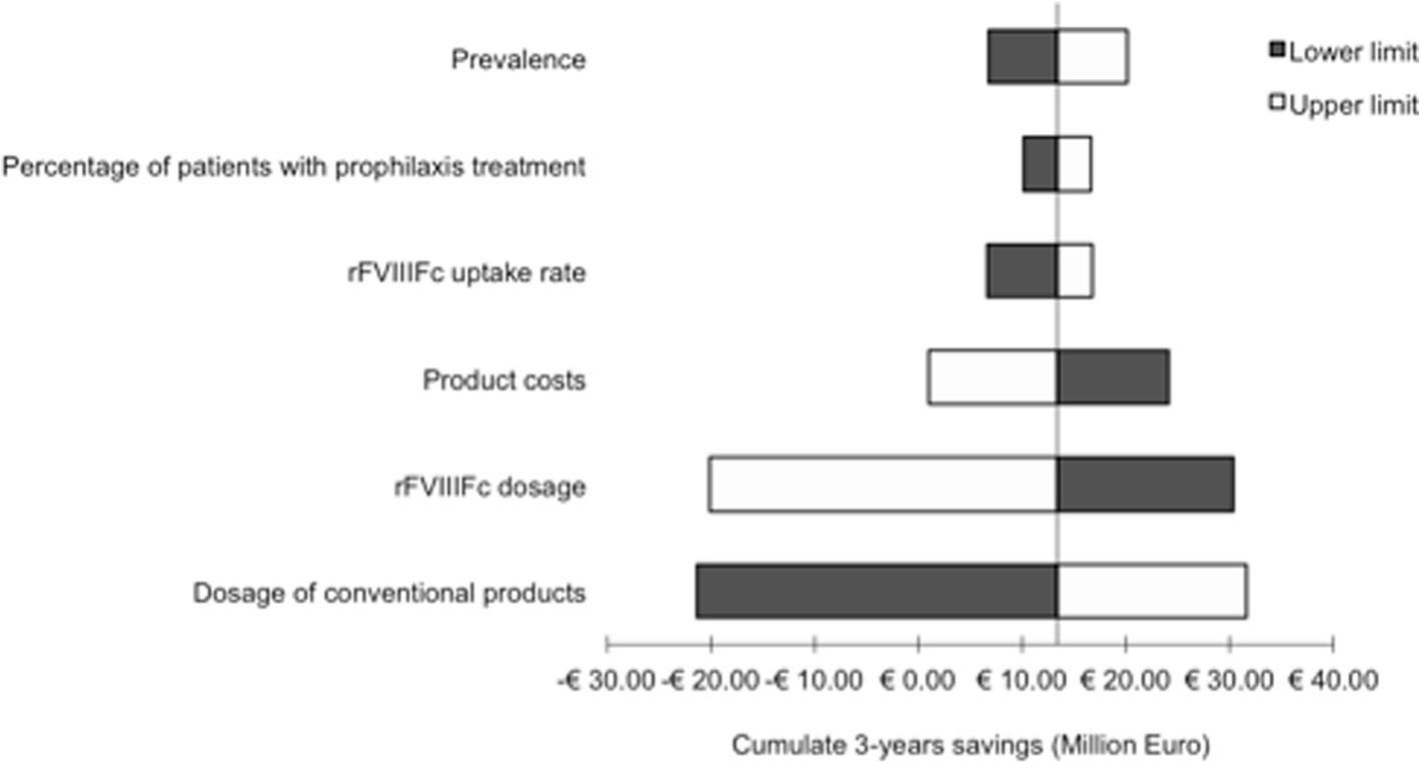
Tornado diagram: results of the one-way sensitivity analysis

The Tornado diagram in Fig. 2 shows the results of the one-way sensitivity analysis.

Per-administration dosage of both rFVIIIFc and the competitors was the major driver of the base-case results.

A 30% dosage decrease for rFVIIIFc resulted in incremental savings, being twice the value estimated in the base-case analysis; conversely, a 30% dosage increase for rFVIIIFc implied increasing costs for the INHS. Decreasing per-administration dosage of conventional products by 30% showed incremental costs of about 30 million Euro, whereas a similar per-administration dosage increase determined incremental savings.

By reducing the prices of comparators by 15%, the savings for the INHS was reduced to about 1 million Euro, while in case of a price decrease of rFVIIIFc savings were estimated to reach 24 million Euro (Fig. 2).

### Scenario analysis 1

When patients with moderate HA were included in the target population, the savings were equal to 18,197,391 Euro. Similarly to the base-case, as the number of patients treated with rFVIIIFc increased, savings rose progressively as shown in the Table 8.

**Table 8:** Scenario analysis

	Overall num. Patients	Num. patients treated with efmoroctocog alfa	Cost CE (Euro)	Cost NE (Euro)	Savings (Euro)	% Savings
Scenario 1	3525	529	755,189,490	736,992,099	18,197,391	2.4%
Scenario 2	4284	474	693,489,851	690,835,256	2,654,595	0.4%

### Scenario analysis 2

This scenario analysis was performed including the possibility of switching treatment regimen from on-demand to prophylaxis treatment for patients treated with plasma-derived therapy and conventional recombinant FVIII; a 5% switch rate was assumed based on the opinion of clinical experts. In this scenario patients treated on-demand were included in the target population in order to assess the savings associated to the switch. The target population considered in this scenario amounted to 4284 patients over 3 years. For the on-demand treatment, based on the opinion of clinical experts, ABR data reported in the A-LONG study [10] and dosage extrapolated from the scientific discussion for the approval of Advate® [29], were used to quantify products’ consumption of conventional treatment. Particularly, the ABR value was assumed to be 33.6, whereas the median dosage per episode and the number of injections were assumed to be 52.15 IU/Kg and 1.34.

In this scenario, savings following the introduction of rFVIIIFc over 3 years were about 2,654,595 Euro.

## Discussion

The economic implication to the NHS of innovative drugs is one of the critical factors contributing to their success and adoption [30]. Despite some criticism regarding application of traditional pharmacoeconomic evaluations applied to rare diseases, the evaluation of the economic impact of innovative drugs could represent a key issue in health policy development also in the context of rare conditions. Indeed, Drummond et al. in a recent review emphasize the importance of the conducting economic evaluations of innovative molecules to allow for more informed treatment evaluation and choice [27].

Policies adopted by OECD countries to encourage development of treatments for orphan diseases contribuited to the proliferation of orphan medicines [31], as well as a steady increase of 5 new orphan drugs per years until 2020 was predicted by Schey C et al. [32]. Moreover, the share of the total pharmaceutical market is predicted to increase until 2016 after which it is expected to level off through 2020 [32]. In this context, the production of evidence related to the economic impact associated to the introduction of novel treatments is essential to inform policy makers and clinical practice, and BIA covers a key role in that framework.

The present analysis outlines the financial impact deriving from the introduction of rFVIIIFc into the market-mix of products for the treatment of HA in Italy. rFVIIIFc has recently been approved by the Italian Medicine Agency as a class A medicine and it is indicated for the treatment and prophylaxis of bleeding episode in patients with haemophilia A (congenital factor VIII deficiency) [33].

Despite BIA is one of the key components for the assessment of the economic impact of a new drug, there is actually a paucity of evidence about this kind of studies [34]; as a strength, to our knowledge this is the first study attempting to evaluate the economic consequences of introducing rFVIIIFc in Italy. The results from the base-case analysis demonstrated a favourable impact resulting in an overall savings of about 13 million Euro over 3 years.

Results are consistent when parameters such as: prevalence, proportion of patients treated with prophylaxis and rFVIIIFc uptake-rate are varied. Conversely, decreasing rFVIIIFc dosage or increasing dosage of conventional products implied increased savings for the INHS. Similarly, when considering a lower price of conventional medicines (and the price of rFVIIIFc remaining constant) the budget impact remained favourable, but savings decreased to about 1 million Euro over the 3-year period.

Even in the scenario analysis considering the possibility of switching from on-demand therapy with conventional products to prophylaxis treatment with rFVIIIFc, the model estimated savings for the INHS, although savings were significantly reduced as compared with the base-case analysis because of the increased costs associated to the prophylaxis regimen.

As a final remark, some of the input data used in the model rely on conservative hypotheses. In particular, similar data about product consumption for the management of spontaneous bleedings were used for conventional products and rFVIIIFc, despite the longer half-life of rFVIIIFc would allow for lower number of administrations.

Our study is limited by the fact that, in presence of a scarcity of data at the time of analysis, some of the inputs used in the model came from single-centre randomized controlled studies; moreover, parameters referred to the different treatments were extrapolated from diverse studies in the absence of head-to-head comparison. These factors thus pose a limit to the possibility of generalizing results from the present analysis.

Haemophilia A is a disabling disorder and treatment requires frequent intravenous injections during prophylaxis and bleeding episodes. According to a recent study conducted in Italy [4], adults with haemophilia have worse quality of life (QoL) than the general population with the physical sphere being the most impaired domain due to problems related to mobility and pain or discomfort. The impact of the disease is also demonstrated to affect both patients and caregivers and the annual social costs of the disease in Italy is estimated to be about 118,000 Euro per person in 2012 [4]. Although haemophilia is not a common disease, the number of people identified with bleeding disorders has increased over the years [35] and this life-long condition place a considerable burden on patients, healthcare systems and society.

Given that the available studies [4, 14, 15] are concordant in outlining that most of HA treatment costs are related to the factor replacement therapy, the introduction of innovative products that may help reducing the overall costs of the therapy while maintaining or even improving the clinical efficacy, could represent an opportunity for the National Health Systems and the society.

Moreover, rFVIIIFc allows a significant reduction in the number of administrations, with decrease median annual bleeding rate [5, 7, 10, 36], thus impacting on the general condition of HA patients, QoL and costs.

Although the compliance to the factor replacement therapy is considered high by clinical experts, the lower number of intravenous injections may increase the adherence to the treatment, particularly in subjects, like children, where compliance may be an issue e.g. due to difficult venous access.

In the scientific literature there is a lack of quantitative analyses measuring the adherence to the treatment, therefore the current model does not take into account the compliance to the treatment. Similarly, costs associated to adverse events or long term effects are not included in the model because of the paucity of data at the time of analysis.

## Conclusion

In conclusion, the model analysed the economic impact in Italy of using Elocta (rFVIIIFc) compared to available conventional FVIIIs for the prophylaxis and treatment of bleeding in children and adults with severe HA. The overall results outlined consistent savings for the INHS at the reimbursed list price of rFVIIIFc. The model considered only direct costs related to the factor consumption, being the main drivers of disease related costs; further evidence related to the long-term efficacy and safety is currently under study and may offer further insights to comprehensively evaluate the economic impact of rFVIIIFc.

## Abbreviations

ABR: Annualized Bleeding Rate
AIFA: Italian Medicines Agency
BIA: Budget Impact Analysis
BIM: Budget Impact Model
CE: Current Environment
FVIII: Factor VIII
HA: Congenital haemophilia A
INHS: Italian National Health System
NE: New Environment
QoL: Quality of life
rFVIII: recombinant factor VIII
rFVIIIFc: recombinant factor VIII-Fc fusion protein
SmPC: Summary of Product Characteristics
